# Development and validation of a risk prediction model for hepatorenal syndrome in hepatic failure patients based on glucose-6-phosphate dehydrogenase and hepatic and renal function biochemical parameters

**DOI:** 10.5937/jomb0-59499

**Published:** 2026-01-06

**Authors:** Hao Liu, Yanmei Lan, Kan Zhang, Tingshuai Wang, Dewen Mao, Minggang Wang

**Affiliations:** 1 Guangxi University of Chinese Medicine, Graduate School, Nanning, Guangxi, 530200, China; 2 The First Affiliated Hospital of Guangxi University of Chinese Medicine, Experimental Center for Science of Traditional Chinese Medicine and Ethnic Medicine, Nanning, Guangxi, 530022, China; 3 The First Affiliated Hospital of Guangxi University of Chinese Medicine, Division of liver disease, Nanning, Guangxi, 530022, China

**Keywords:** hepatorenal syndrome, hepatic failure, G6PD, diagnostic model, liver and kidney function, hepatorenalni sindrom, insuficijencija jetre, G6PD, dijagnostički model, funkcija jetre i bubrega

## Abstract

**Background:**

This study aimed to develop and validate a novel risk prediction model for hepatorenal syndrome (HRS) in hepatic failure (HF) patients by integrating glucose-6-phosphate dehydrogenase (G6PD) activity with conventional hepatic and renal function biochemical parameters, thereby enhancing early HRS detection beyond the limitations of traditional indicators.

**Methods:**

We performed a retrospective analysis of 264 HF patients (82 with HRS, 182 without HRS) hospitalized between July 2020 and July 2022. G6PD levels and standard hepatic/renal function biochemical parameters (ALT, AST, TBil, GGT, BUN, Scr, UA, and CysC) were assessed. Key predictors were identified via Least Absolute Shrinkage and Selection Operator (LASSO) regression, and a multivariate logistic regression model was developed. Model performance was evaluated using receiver operating characteristic (ROC) analysis, with internal validation conducted through a 70:30 training-validation split.

**Results:**

HRS patients exhibited significantly lower G6PD activity than non-HRS HF controls (P &lt; 0.05). While G6PD alone showed moderate predictive value (AUC = 0.742; sensitivity 59.76%, specificity 79.12%), the composite model integrating G6PD, GGT, UA, Scr, and CysC demonstrated markedly improved discrimination, achieving AUCs of 0.960 (95%CI: 0.931-0.990) in the training cohort and 0.957 (95%CI: 0.913-1.000) in the validation cohort with both sensitivity and specificity outperforming individual indicators. The derived risk equation was Combined testing Youden = -17.038 + -0.116 x G6PD + 0.102 x GGT + 0.016 x UA + 0.040 x Scr + 3.760 x CysC.

**Conclusions:**

The integration of G6PD with hepatic and renal function biochemical parameters significantly enhances HRS risk stratification in HF patients. This validated tool offers superior sensitivity and specificity for the early identification of HRS.

## Abstract (sr)

**Uvod:**

Cilj ove studije bio je razvoj i validacija novog modela predviđanja rizika za hepatorenalni sindrom (HRS) kod pacijenata sa insuficijencijom jetre (SF) integrisanjem aktivnosti glukoza-6-fosfat dehidrogenaze (G6PD) sa konvencionalnim biohemijskim parametrima funkcije jetre i bubrega, čime se poboljšava rano otkrivanje HRS-a izvan ograničenja tradicionalnih indikatora.

**Metode:**

Sproveli smo retrospektivnu analizu 264 pacijenta sa srčanom insuficijencijom (82 sa HRS, 182 bez HRS) hospitalizovanih između jula 2020. i jula 2022. godine. Procenjeni su nivoi G6PD i standardni biohemijski parametri funkcije jetre/bubrega (ALT, AST, TBil, GGT, BUN, Scr, UA i CysC). Ključni prediktori su identifikovani pomoću regresije najmanjeg apsolutnog smanjenja i operatora selekcije (LASSO), i razvijen je multivarijantni logistički regresioni model. Performanse modela su procenjene korišćenjem ROC analize, sa internom validacijom sprovedenom putem podele obuke i validacije u odnosu 70:30.

**Rezultati:**

Pacijenti sa HRS-om pokazali su značajno nižu aktivnost G6PD u odnosu na kontrolnu grupu sa HF bez HRS-a (P &lt; 0,05). Dok je G6PD sam po sebi pokazao umerenu prediktivnu vrednost (AUC = 0,742; osetljivost 59,76%, specifičnost 79,12%), kompozitni model koji integriše G6PD, GGT, UA, Scr i CysC pokazao je značajno poboljšanu diskriminaciju, postižući AUC od 0,960 (95%CI: 0,931-0,990) u kohorti za obuku i 0,957 (95%CI: 0,913-1,000) u kohorti za validaciju, pri čemu su i osetljivost i specifičnost nadmašile pojedinačne indikatore. Izvedena jednačina rizika bila je Kombinovano testiranje Youden = -17,038 + -0,116 x G6PD + 0,102 x GGT + 0,016 x UA + 0,040 x Scr + 3,760 x CysC.

**Zaključak:**

Integracija G6PD sa biohemijskim parametrima funkcije jetre i bubrega značajno poboljšava stratifikaciju rizika od HRS kod pacijenata sa srčanom insuficijencijom. Ovaj validirani alat nudi superiornu osetljivost i specifičnost za ranu identifikaciju HRS.

## Introduction

Hepatorenal syndrome (HRS), a severe and life-threatening complication of hepatic failure (HF), is characterized by progressive functional renal impairment and is pathophysiologically associated with systemic hemodynamic disturbances, profound splanchnic vasodilation, and compensatory renal vasoconstriction [1]. Clinical epidemiology reveals a striking incidence of HRS, affecting 20%-40% of acute HF patients and 10%-20% of those with decompensated cirrhosis [2]. The development of HRS portends a dismal prognosis, with untreated cases demonstrating a precipitous decline in survival, evidenced by a 28-day mortality rate exceeding 50% [3]. While recent years have witnessed advancements in HRS management strategies, the critical challenge of early detection persists [4]. Current diagnostic paradigms, reliant on exclusion criteria and late-appearing biomarkers like serum creatinine, inevitably delay therapeutic intervention, thereby compromising patient outcomes [5]. Therefore, exploring sensitive and specific early biomarkers and constructing robust risk prediction models are urgently needed to enhance clinical decision-making in HF management.

At the molecular level, glucose-6-phosphate dehydrogenase (G6PD) serves as the pivotal rate-limiting enzyme in the pentose phosphate pathway, governing the production of NADPH through its catalysis of glucose-6-phosphate oxidation. This fundamental biochemical role positions G6PD as a crucial regulator of cellular redox balance and oxidative stress defense mechanisms [6]. Emerging evidence implicates dysregulated G6PD activity in the pathogenesis of diverse hepatic disorders, including nonalcoholic fatty liver disease and viral hepatitis [7]. In the context of HF, the catastrophic failure of hepatic metabolic function precipitates a marked reduction in G6PD activity, which in turn amplifies oxidative stress and mitochondrial dysfunction, creating a vicious cycle that drives multiorgan injury [8]. Of particular clinical relevance, oxidative stress exerts dual deleterious effects: not only does it directly impair hepatic regenerative capacity, but it also potentiates HRS progression through induction of renal tubular epithelial apoptosis and disruption of renal vascular endothelial function [9]. While G6PD deficiency has been independently associated with renal functional decline in chronic kidney disease [10], its precise contribution to HF-associated HRS pathogenesis remains to be elucidated.

Contemporary approaches to HRS risk stratification, both in domestic and international research landscapes, predominantly employ isolated organ function parameters or composite clinical scoring systems [e.g., Model for End-Stage Liver Disease (MELD)]. However, these conventional models demonstrate suboptimal specificity for HRS prediction and lack incorporation of biomarkers that directly reflect renal tubular damage [11] [12]. In light of this, our study implements a multicenter prospective cohort design to comprehensively evaluate the temporal dynamics of G6PD in conjunction with hepatic and renal biochemical parameters, with the ultimate goal of developing a novel risk prediction algorithm for HRS in HF patients. This investigation represents the first concerted effort to incorporate G6PD—an emerging metabolic regulator—into the predictive paradigm for HF-related HRS, thereby transcending the constraints of traditional models that focus narrowly on glomerular filtration parameters. The findings hold substantial promise for advancing early HRS detection, enabling precision therapeutic interventions, and uncovering novel mechanistic insights, all of which may ultimately contribute to reduced HF mortality and more efficient allocation of critical healthcare resources.

## Materials and methods

### Study population

**Figure 1:**
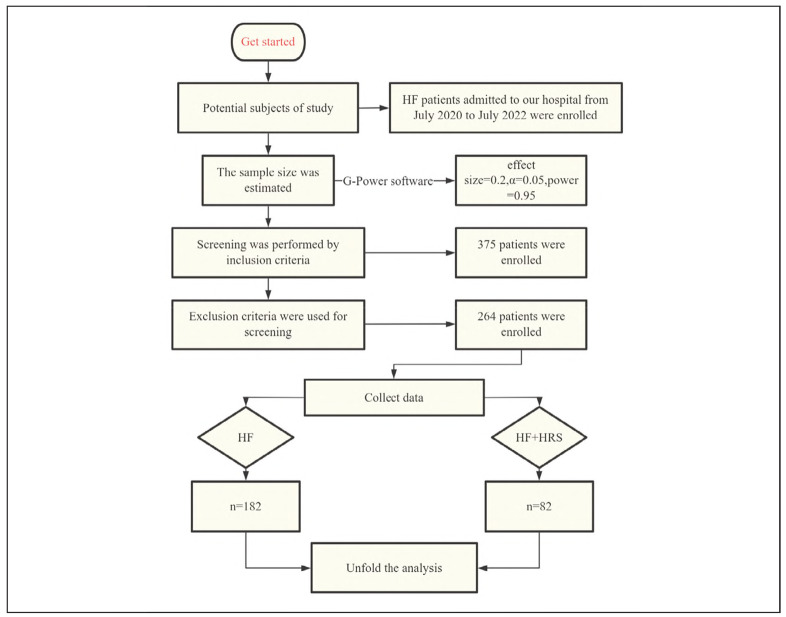
The main flow of this study.

We conducted a retrospective cohort study involving patients diagnosed with HF who were admitted to our institution between July 2020 and July 2022. The sample size was determined using G-Power software (effect size = 0.2, α = 0.05, power = 0.95), yielding a minimum required sample of 262 subjects. After applying the predefined inclusion and exclusion criteria, 264 eligible participants were enrolled in the final analysis. All data were anonymized using unique patient IDs, in compliance with the GDPR (General Data Protection Regulation) and HIPAA (Health Insurance Portability and Accountability Act). The cohort comprised 82 patients with concurrent HRS and 182 patients with HF alone. Ethical approval for this study was obtained from the Institutional Review Board of the First Affiliated Hospital of Guangxi University of Chinese Medicine (Approval No. 2020-046-02), and all procedures adhered to the ethical principles outlined in the Declaration of Helsinki. Given the retrospective design of the study, the requirement for informed consent was waived. Figure 1 presents the participant selection flowchart and study design schematic.

### Inclusion and exclusion criteria

Inclusion criteria: Confirmed diagnosis of HF and/or HRS according to established diagnostic criteria [13] [14]; Age ≥18 years; Availability of complete medical records. Exclusion criteria: Pre-existing organic renal disease or obstructive uropathy; Extrahepatic malignancies; Recent use of nephrotoxic medications; Severe immunosuppression (including drug-induced immunosuppression or immunodeficiency disorders such as advanced tuberculosis or acquired immune deficiency syndrome); Comorbid severe systemic illnesses or major psychiatric disorders; Current pregnancy or lactation status.

### Laboratory procedures

Fasting venous blood samples were collected from patients upon admission. Following a 30-minute incubation at room temperature, samples were centrifuged at 3000 rpm/min for 15 minutes to obtain plasma. G6PD activity was quantified spectrophotometrically by measuring the rate of NADPH production. Erythrocytes were lysed and incubated with a reaction mixture containing glucose-6-phosphate, NADP+, and buffer solution. NADPH generation was monitored by absorbance at 340 nm, with enzyme activity expressed as units per gram of hemoglobin (U/g Hb). Quality control: Bio-Rad Lyphochek Immunoassay Plus quality control (level 1/2) was used and validated daily before detection. The spectrophotometer was calibrated monthly using calibrators provided by the manufacturer. CLSI (Clinical and Laboratory Standards Institute) EP5-A3 document was followed to ensure intra-assay CV<5% and interassay CV<8%.

Liver and kidney function [Alanine Aminotransferase (ALT), Aspartate Aminotransferase (AST), Total Bilirubin (TBil), Gamma-Glutamyl Transferase (GGT), Blood Urea Nitrogen (BUN), Serum Creatinine (Scr), Uric Acid (UA), and Cystatin C (CysC)]: Serum samples were detected by automatic biochemical analyzer (Beckman AU5800), and the reaction kinetics and concentration were automatically monitored by the instrument. Quality control: BioRad CPS1-CPS3 quality control serum, measured daily at double levels. According to ISO 15189: 2012 standard, the linear range of the calibration curve covered the clinical requirements.

### Data collection

The study recorded G6PD enzymatic activity measurements, baseline characteristics (age, sex, disease duration, etc.), and hepatic and renal function parameters (ALT, a St, TBil, GGT, BUN, Scr, UA, and CysC).

### Statistical methods

Analyses were conducted using SPSS 25.0. Categorical variables are presented as frequencies [n (%)] with between-group comparisons made using χ^2^ tests. Continuous variables were assessed for normality using the Shapiro-Wilk test. Normally distributed data are reported as mean ± standard deviation (χ±s) and analyzed with independent t-tests; non-normal data are presented as median (interquartile range) with Mann-Whitney U tests for comparisons. Predictor variables were selected through Least Absolute Shrinkage and Selection Operator (LASSO) regression [Lasso regression employed 10-fold cross-validation with the 'glmnet' package in R, selecting λ via the 'minimum criteria' (λ=0.45) corresponding to the smallest mean squared error (MSE) plus one standard error (λ + SE=0.772)], with subsequent logistic regression modeling for risk prediction. Covariates with P<0.1 in univariate analysis (age, ascites, infection) were included in the multivariate model to adjust for confounding. Model discrimination was evaluated using receiver operating characteristic (ROC) curve analysis, where area under the curve (AUC) values approaching 1.0 indicate superior diagnostic accuracy. The corresponding cut-off value was selected according to the largest Youden index, and the corresponding diagnostic sensitivity and specificity were recorded. Statistical significance was defined as P < 0.05.

## Results

### Comparison of baseline characteristics

Analysis of baseline characteristics and hepatic/renal function parameters revealed no significant intergroup differences in sex, disease duration, or smoking/alcohol consumption status (P > 0.05). However, patients with HF+HRS demonstrated significantly elevated values in age, ALT, AST, TBil, GGT, BUN, Scr, UA, and CysC compared to HF patients (P < 0.05). Notably, the prevalence of ascites and spontaneous bacterial peritonitis was significantly higher among HF+HRS patients (P < 0.05, Table 1).

**Table 1:** Comparison of baseline data, liver and kidney function between HF patients and HF+HRS patients.

Factors	HF patients<br>(n = 182)	HF+HRS patients<br>(n= 82)	χ^2^ (or t) values	P values
Gender			0.270	0.603
male	146 (80.22)	68 (82.93)		
female	36 (19.78)	14 (17.07)		
Age (years)			5.778	0.016
<55	111 (60.99)	37 (45.12)		
≥55	71 (39.01)	45 (54.88)		
Mean age	54.39±6.29	57.10±7.72	3.009	0.003
Drinking			0.101	0.751
no	158 (86.81)	70 (85.37)		
yes	24 (13.19)	12 (14.63)		
Smoking			2.332	0.128
no	170 (93.41)	72 (87.80)		
yes	12 (6.59)	10 (12.20)		
Liver function				
ALT (U/L)	41.20±13.3 9	46.68±12.30	3.157	0.002
AST (U/L)	80.42±22.34	143.06±30.24	18.804	<0.001
TBil (μmol/L)	112.28±31.84	121.15±30.55	2.120	0.035
GGT (U/L)	70.13±19.28	114.79±23.87	16.141	<0.001
Renal function				
BUN (mmol/L)	4.43±0.96	4.86±1.25	3.046	0.003
Scr (μmol/L)	73.60±16.66	83.02±20.04	3.984	<0.001
UA (μmol/L)	204.37±35.00	229.04±34.27	5.332	<0.001
CysC (mg/L)	1.19±0.22	1.35±0.27	5.186	<0.001
Ascitic fluid			7.581	0.006
no	160 (87.91)	61 (74.39)		
yes	22 (12.09)	21 (25.61)		
Gastrointestinal bleeding			2.172	0.141
no	173 (95.05)	74 (90.24)		
yes	9 (4.95)	8 (9.76)		
Bacterial peritonitis			9.029	0.003
no	152 (83.52)	55 (67.07)		
yes	30 (16.48)	27 (32.93)		
Infection			0.190	0.663
no	179 (98.35)	80 (97.56)		
yes	3 (1.652)	2 (2.44)		

### G6PD activity analysis

**Figure 2:**
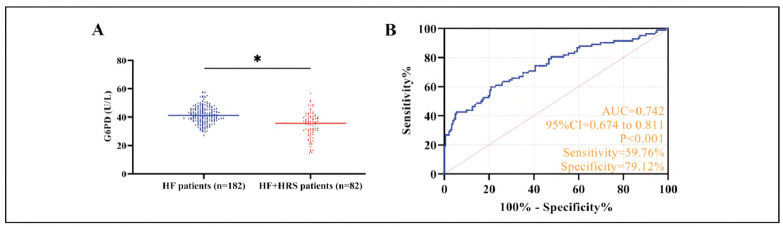
Analysis of clinical significance of G6PD in HF+HRS.<br>A: Comparison of G6PD between patients with HF and those with HF + HRS, *P < 0.05. B: ROC curve of G6PD for the diagnosis of HRS in HF patients.

Serum G6PD activity was significantly reduced in HF+HRS patients compared to HF controls (P < 0.05). ROC curve analysis identified a G6PD cutoff value of 37.02 U/L (G6PD<37.02 U/L) for HRS diagnosis in HF patients, yielding 59.76% sensitivity and 79.12% specificity (P < 0.05). The AUC of 0.736 suggests moderate diagnostic utility (Figure 2).

### Indicator screening for the HF+HRS risk model

**Figure 3:**
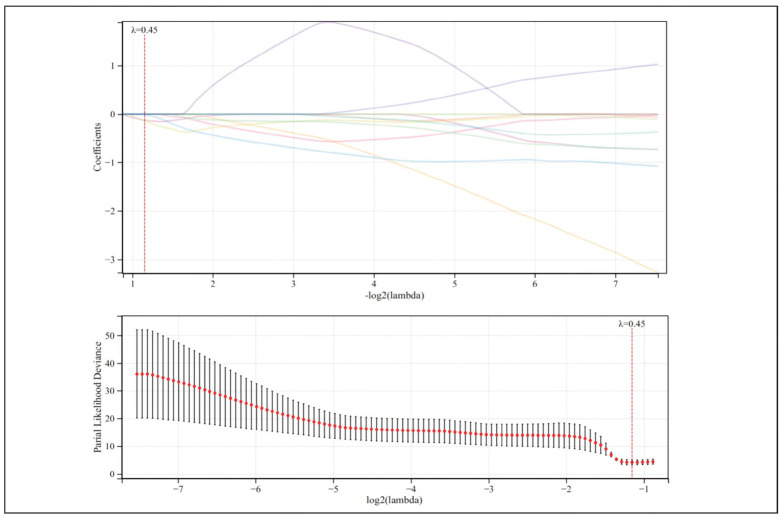
LASSO analysis to screen the relevant factors of the risk model for HF complicated with HRS.

Variables showing significant differences in univariate analysis (Table 1), along with G6PD, were subjected to LASSO regression with cross-validation for optimal λ selection. The analysis identified λ = 0.45 as minimizing the mean squared error (MSE), with λ + 1 standard error at 0.772. As illustrated in Figure 3, as log (λ) increased, four indicators—G6PD, GGT, UA, Scr, and CysC—were ultimately retained.

### Development of the HF+HRS risk prediction model

A binary logistic regression model was constructed with HRS development as the outcome variable (HF = 1, HF+HRS = 2), incorporating G6PD, GGT, UA, Scr, and CysC as covariates. Multi-collinearity was assessed using variance inflation factors (VIF). All VIF values were <3.0, indicating no significant collinearity. Based on the analysis results, the combined detection formula was derived as Combined testing_Youden_ = -17.038 + -0.116 × G6PD + 0.102 × GGT + 0.016 × UA + 0.040 × Scr + 3.760 × CysC (Table 2).

**Table 2:** Logistic regression analysis of risk factors.

	β	S.E.	Wals	Sig.	Exp (β)	95%CI
GGT	0.102	0.016	42.918	<0.001	1.108	1.074-1.142
Scr	0.040	0.016	6.536	0.011	1.041	1.00-1.074
UA	0.016	0.008	4.473	0.034	1.016	1.001-1.032
GysC	3.760	1.149	10.711	0.001	2.932	1.518-4.973
G6PD	-0.116	0.039	8.694	0.003	0.891	0.825-0.962
Constant	-17.038	3.599	22.416	<0.001	-	-

### Validation of the HF+HRS risk model

**Figure 4:**
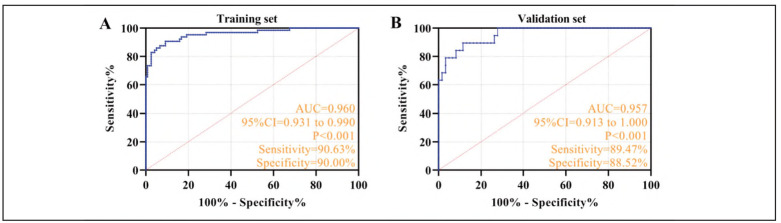
Validation of the effect of the risk model for HF patients complicated with HRS. A: ROC curve of the training set. B: ROC curve of the validation set.

The cohort was randomly divided into training (70%, n = 187) and validation (30%, n=80) sets using computer-generated randomization. ROC analysis demonstrated robust predictive accuracy, with the training set achieving an AUC of 0.960 (95%CI: 0.931-0.990), 90.63% sensitivity, and 90.00% specificity. Comparable performance was observed in the validation set (AUc = 0.957; 95%CI: 0.0.913-1.000); sensitivity=89.47%; specificity=88.52%) (Figure 4), which has an excellent prediction effect.

### Visualization of the HF+HRS risk model

**Figure 5:**
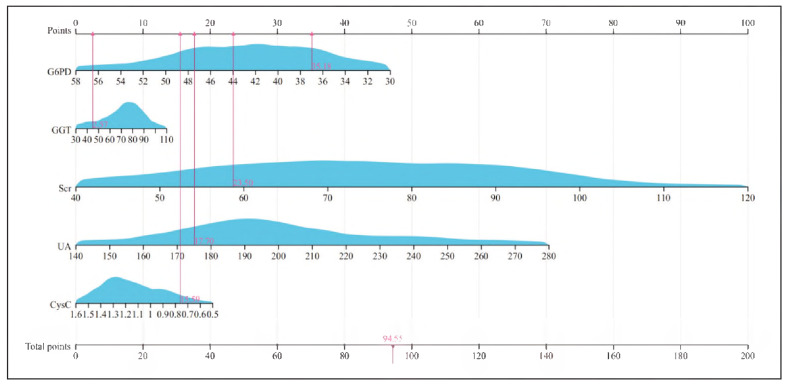
Visual processing of the risk model.

The nomogram was constructed using the 'rms' package in R, assigning weights to each predictor based on β coefficients (range: 0.016-3.760). Total scores were converted to probabilities using the formula: Probability = 1/(1 +exp(-(Intercept+ΣβX))).The aggregate risk score, calculated by summing individual component scores, showed a positive correlation with HRS development probability in HF patients (Figure 5).

## Discussion

This study establishes serum G6PD as a sensitive metabolic biomarker that demonstrates a strong correlation with the risk of HRS development in HF patients. Notably, our integrated risk prediction model incorporating G6PD with standard hepatic and renal biochemical parameters exhibited robust performance in HRS risk stratification, offering novel clinical perspectives for early HRS detection and management [13] [14].

As the rate-limiting enzyme of the pentose phosphate pathway, G6PD catalyzes the oxidation of glucoses-phosphate to generate NADPH, thereby serving as a crucial regulator of cellular redox balance and oxidative stress defense mechanisms [15]. In HF patients, the collapse of hepatic metabolic function may lead to a significant decline in G6PD activity, resulting in compromised NADPH biosynthesis [16]. Given that NADPH represents the principal cellular reducing equivalent, its depletion precipitates a cascade of oxidative damage, including mitochondrial impairment, lipid peroxidation, and protein oxidative damage [17]. These pathophysiological alterations not only compromise hepatic regenerative potential but also predispose to renal dysfunction through multiple interconnected pathways. Our mechanistic analysis suggests three predominant mechanisms linking G6PD deficiency to HRS pathogenesis: (1) Renal tubular epithelial cell apoptosis: Oxidative stress mediates the activation of pro-apoptotic signaling pathways (e.g., JNK/p38 MAPK), inducing apoptosis of renal tubular epithelial cells [18]. (2) Renal vascular endothelial dysfunction: Impaired NADPH-dependent endothelial nitric oxide synthase (eNOS) function reduces nitric oxide (NO) production, exacerbating renal vasoconstriction [19]. (3) Inflammatory cascade: Oxidative stress promotes the release of proinflammatory factors (e.g., IL-6, TNF-α), activating inflammatory signaling pathways (e.g., NF-κB) in renal tissue, further damaging renal parenchyma [20]. Our clinical data revealed significantly depressed G6PD activity in HF+HRS patients compared to HF controls (AuC = 0.742 for HRS prediction), underscoring its potential as a metabolic indicator of renal compromise in HRS. These findings corroborate previous observations by Shi Z et al. [21] demonstrating a close association between G6PD deficiency and accelerated renal function decline in cirrhosis (HR=1.89, P=0.003). Preclinical evidence from murine models provides further validation, with G6PD-knockout animals exhibiting exacerbated renal tubular necrosis and oxidative tissue damage following hepatic ischemia-reperfusion injury [22], thereby reinforcing the pivotal role of G6PD in mediating hepatorenal pathophysiology.

Although this study demonstrates an association between diminished G6PD activity and HRS risk, the standalone utility of G6PD measurement presents notable limitations. For example, G6PD activity exhibits variability due to genetic polymorphisms (e.g., G6PD deficiency), infections, and pharmacological interventions (e.g., sulfonamide use) [23], potentially yielding false-positive or false-negative results due to these confounding variables. Moreover, as a static measurement, G6PD activity provides only a snapshot of redox status and fails to capture the dynamic progression of hepatic and renal dysfunction in HRS. While the pathogenesis of HRS is known to involve complex interactions among systemic hemodynamic disorders, inflammatory cascades, and metabolic dysregulation—a multifaceted process that cannot be adequately represented by any single biomarker.

To address this issue, this study attempted to improve the clinical applicability of the model by integrating G6PD with conventional hepatic and renal function biochemical parameters to distinguish HRS-specific injury from other confounding factors. Our findings revealed significantly elevated levels of age, ALT, AST, TBil, GGT, BUN, Scr, UA, and CysC in HF + HRS patients compared to HF controls, aligning with established HRS pathophysiology [24]. Through Lasso regression optimization, we constructed a predictive model incorporating G6PD, GGT, UA, Scr, and CysC that achieved an AUC of 0.942 for hRs detection. This robust performance suggests that renal impairment in HF patients stems not merely from hemodynamic alterations but also from worsening liver function. For example, hyperbilirubinemia (elevated TBil) can exacerbate kidney injury by inducing oxidative stress and mitochondrial dysfunction in renal tubules [25]. Meanwhile, elevated GGT, indicative of hepatic damage, may compromise detoxification capacity, leading to nephrotoxic endotoxin accumulation and indirect kidney injury [26]. This multidimensional biomarker approach addresses critical limitations of conventional scoring systems (e.g., MELD), which focus predominantly on glomerular function. By providing a more comprehensive assessment of HRS pathophysiology, our model facilitates earlier and more accurate identification of high-risk patients, allowing for timely implementation of targeted interventions such as enhanced monitoring, pharmacologic optimization, and supportive care strategies. Such precision medicine approaches may ultimately improve clinical outcomes while optimizing healthcare resource utilization.

According to the results of this study, in the future, this model can be used to judge the pathological progress of HF patients and provide accurate objective guidance for the prevention of HRS, which may greatly improve the prognosis of HF patients. However, as a single-center retrospective study, our research has inherent limitations, including a modest sample size and potential selection bias. In addition, the lack of dynamic G6PD level monitoring precludes definitive conclusions regarding its temporal relationship with HRS progression. Furthermore, the mechanistic insights remain incomplete due to insufficient exploration of G6PD's association with key inflammatory mediators (e.g., IL-6, TNF-α). Future investigations should employ multicenter prospective cohort designs, complemented by single-cell sequencing or renal histopathological analyses, to better delineate the molecular mechanisms underlying G6PD release. Additionally, therapeutic targeting of G6PD-associated pathways (e.g., TLR4/NF-κB) warrants further exploration as a potential strategy for HRS management.

## Conclusion

G6PD contributes significantly to the pathogenesis of HF-associated HRS through redox homeostasis regulation. However, its standalone use suffers from nonspecificity and mechanistic limitations. By developing an integrated predictive model combining G6PD with hepatic/renal function parameters, our study enhances early HRS detection and offers a clinically actionable tool for timely intervention.

## Dodatak

### Availability of data and materials

The data that support the findings of this study are available from the corresponding author upon reasonable request.

### Funding

This study was supported by the Guangxi Key Research and Development Program (GuiKe AB25069020); Natural Science Foundation of China (No.82360912; No.82274434), Guangxi Postgraduate Education Innovation Program Project (NO. YCSW2025456), and National Traditional Chinese Medicine Inheritance and Innovation Center Construction Project.

### Conflict of interest statement

All the authors declare that they have no conflict of interest in this work.
